# Circulating Permeability Factors in Primary Focal Segmental Glomerulosclerosis: A Review of Proposed Candidates

**DOI:** 10.1155/2016/3765608

**Published:** 2016-04-21

**Authors:** Eva Königshausen, Lorenz Sellin

**Affiliations:** Department of Nephrology, University Hospital, Heinrich-Heine University, Moorenstrasse 5, 40225 Duesseldorf, Germany

## Abstract

Primary focal segmental glomerulosclerosis (FSGS) is a major cause of the nephrotic syndrome and often leads to end-stage renal disease. This review focuses on circulating permeability factors in primary FSGS that have been implicated in the pathogenesis for a long time, partly due to the potential recurrence in renal allografts within hours after transplantation. Recently, three molecules have been proposed as a potential permeability factor by different groups: the soluble urokinase plasminogen activator receptor (suPAR), cardiotrophin-like cytokine factor-1 (CLCF-1), and CD40 antibodies. Both CLCF-1 and CD40 antibodies have not been validated by independent research groups yet. Since the identification of suPAR, different studies have questioned the validity of suPAR as a biomarker to distinguish primary FSGS from other proteinuric kidney diseases as well as suPAR's pathogenic role in podocyte damage. Researchers have suggested that cleaved molecules of suPAR have a pathogenic role in FSGS but further studies are needed to determine this role. In future studies, proposed standards for the research of the permeability factor should be carefully followed. The identification of the permeability factor in primary FSGS would be of great clinical relevance as it could influence potential individual treatment regimen.

## 1. Introduction

Primary and secondary focal segmental glomerulosclerosis (FSGS) are a major cause of nephrotic syndrome in the United States and often lead to end-stage renal disease (ESRD) [1]. FSGS is diagnosed and classified from renal biopsies [2, 3]. Injury of podocytes initiates the disease process of FSGS, leading to the classic focal distribution of sclerosis with a segmental pattern within the glomeruli [4]. Clinically, patients present with an abrupt onset of proteinuria, hypoalbuminemia, and edema. Causes of FSGS are heterogeneous and this paper will only focus on the pathogenesis of primary FSGS, in particular on circulating permeability factors in primary FSGS.

Primary FSGS is diagnosed if gene mutations and other causes of FSGS (glomerular hyperfiltration, virus infection, drugs, etc.) have been ruled out. Primary FSGS accounts for approximately 40% of idiopathic nephrotic syndromes. Even though the idiopathic nephrotic syndrome is a rare disease with an incidence of 7 per 1 million [5], it often leads to severe renal impairment and ESRD and the response to immunosuppressive therapy is poor.

The etiology of primary FSGS is still unknown. However, circulating permeability factors have been implicated in the pathogenesis of FSGS for a long time due to the following observations [6]. First, proteinuria recurs in patients with primary FSGS after renal transplantation in more than 30% of cases [7]. Interestingly, this proteinuria may develop within hours after transplantation and some patients benefit from plasmapheresis [8, 9]. Second, infusion of plasma from FSGS patients causes proteinuria in rats [10–12]. In a model for testing glomerular permeability, sera from some FSGS patients also increased permeability to albumin in isolated rat glomeruli [13]. Third, transmission of a potential permeability factor from a pregnant woman with primary FSGS to her newborn infant has been published. The infant presented with transient proteinuria [14]. Lastly, a patient with primary FSGS who received a kidney transplant from his healthy sister developed proteinuria and a decline of renal function shortly after transplantation. FSGS recurrence was confirmed by renal biopsy and, despite treatment with plasmapheresis, the transplant did not regain function. Two weeks after transplantation, the allograft was removed and transplanted into another recipient who had ESRD due to diabetic nephropathy. Proteinuria declined rapidly and the histological lesions disappeared on biopsy samples. Kidney function remained stable for at least 8 months after transplantation [15].

Taken together, these observations strongly suggest a causative role of one or more circulating permeability factor(s) in recurrent primary FSGS.

## 2. Circulating Permeability Factors in Primary FSGS

A recent review on nephrotic syndromes described among other things the historical perspectives of the permeability factors identification in idiopathic nephrotic syndrome [6]. Many investigators have used different models to test permeability factors and comparisons amongst these studies are therefore difficult due to the lack of strict criteria of how putative disease-causing permeability factors are defined. Maas et al. have now proposed criteria to define pathogenic circulating factors in MCD and FSGS [6]. We agree with the authors in the attempt to standardize these criteria, even though as a result research in this field will become much more complicated.

The molecular characteristics of permeability factors have been derived from observations that the active fraction of sera from patients with FSGS precipitates in 70–80% ammonium sulfate solution independent of the immunoglobulin fraction. The putative permeability factor(s) are bound to protein A and had a molecular size between 30 and 50 kDa [13]. Immunoabsorption with a protein A column reduced proteinuria in a patient with recurrent FSGS [16]. When the 30–50 kDa fraction was infused into rats, proteinuria developed [17]. In addition, it was proposed that the circulating factor in FSGS interacts with the glycocalyx of the podocytes. To prevent this interaction, galactose was tested and had a high affinity to the active fraction of FSGS sera that was greater than 30 kDa [18]. Furthermore, oral galactose caused a decrease in the active fraction of FSGS serum in a patient with recurrent, plasmapheresis resistent FSGS. Harris et al. reported that FSGS sera increased protease activated receptor-1 mediated phosphorylation of the vasodilator stimulated protein (VASP) in human podocytes, indicating a pathological role for circulating proteases in FSGS [19]. Recently, a novel* in vitro* assay to test the probability of FSGS recurrence was published [20]. Sera from patients with FSGS recurrence disrupted podocyte focal complexes imaged by immunofluorescence.

Recently, three candidate proteins have been proposed to be the circulating factor in FSGS (Table 1). These will be reviewed in more detail.

### 2.1. Soluble Urokinase Plasminogen Activator Receptor (suPAR)

Urokinase plasminogen activator receptor (uPAR) is a cell membrane glycosylphosphatidylinositol- (GPI-) anchored protein expressed in many cell types, for example, immune cells [21–23], endothelial cells [24], tumor cells [25], tubular epithelial cells [26], and podocytes [27]. uPAR is composed of three domains (*D*_I_, *D*_II_, and *D*_III_) that bind to their ligand urokinase plasminogen activator (uPA). Through interaction with transcellular receptors, such as integrins, uPAR promotes cell migration, proliferation, and survival [28].

Through cleavage of uPAR from its GPI-anchor, the soluble urokinase plasminogen activator receptor (suPAR) is released. Further cleavage between the *D*_I_ and *D*_II_/*D*_III_ domains of suPAR generates other cleaved suPAR fragments. suPAR and uPAR are heavily glycosylated proteins. Depending on the amount of glycosylation and the size of the cleaved proteins, suPAR's size ranges from 25 to 50 kDa. suPAR can be detected in plasma, serum, urine, and other body fluids. In healthy individuals, suPAR is present at low levels regulating neutrophil trafficking and stem cell mobilization [29]. Infections and inflammatory diseases lead to an increase in suPAR levels indicating a role as an acute phase reactant [30–34].

#### 2.1.1. suPAR as Biomarker

Recently, suPAR has emerged as a biomarker in different disease conditions. For example, suPAR concentrations were associated with increased risk of cardiovascular events in the general population [35, 36]. In patients with myocardial infarction (MI), suPAR levels predicted recurrent MI and mortality [37, 38]. In addition, suPAR concentration correlated with mortality in critically ill patients beyond validated score systems [34, 39]. Patients with chronic kidney disease (CKD) are known to be at increased risk of cardiovascular events. In line with the observations in the general population, suPAR was associated with mortality and new-onset cardiovascular disease in a mild-moderate CKD cohort [40].

Several studies have described an inverse correlation of suPAR levels with the estimated glomerular filtration rate (eGFR) [41–44]. Recently, Hayek et al. investigated the role of plasma suPAR levels and the incidence of CKD in a prospective cohort study of patients with cardiovascular disease [45]. In this cardiovascular patient cohort, suPAR levels were independently associated with the decline in eGFR and the development of CKD (defined as eGFR <60 mL/min/1.73 m^2^). In addition, suPAR levels were positively correlated with the incidence of proteinuria. However, proteinuria data from this study needs to be interpreted with caution, as the absolute patient numbers with proteinuria were low and proteinuria was diagnosed only semiquantitatively via urine dipstick.

#### 2.1.2. suPAR in FSGS

The first evidence that uPAR plays a role in podocyte biology was published by Wei et al. in 2008 [27]. Quite recently, the same group published an article in which they identified suPAR as a possible causal factor in FSGS [46]. The authors found increased concentrations of suPAR in patients with FSGS. However, patients with minimal change disease (MCD), membranous nephropathy (MN), and preeclampsia did not display a significant elevation of suPAR levels. The highest suPAR concentrations were found in patients with recurrent FSGS. In addition, suPAR levels correlated with the presence but not with the level of proteinuria. The authors also proposed a pathological cut-off for suPAR. Levels of 3000 pg/mL and above were present in two-thirds of FSGS patients, however much less in other proteinuric kidney diseases. To prove the causal impact of suPAR, Wei et al. performed cell culture and mouse experiments that are described in more detail below.

#### 2.1.3. Does suPAR Discriminate Primary FSGS from Other Proteinuric Kidney Diseases?

In the FSGS CT (70 adults) and Podonet (94 children) cohort, Wei et al. tested suPAR levels in patients with primary FSGS using the proposed cut-off level of 3000 pg/mL [47]. The three major findings of this study were that suPAR levels were elevated in 84.3% (CT cohort) or 55.3% (Podonet cohort) of patients with primary FSGS. Second, suPAR levels did not correlate with inflammation measured by C-reactive protein (CRP) values and, third, mycophenolate mofetil (MMF) therapy was associated with a decline in suPAR levels. There was an inverse correlation of suPAR levels with eGFR. Interestingly, female patients had higher suPAR levels in both cohorts. Li et al. confirmed in their cohort (109 primary FSGS, 20 MCD, 22 MN, and 96 healthy controls) that suPAR levels were elevated in about half of their patients with FSGS and could therefore discriminate between FSGS and other proteinuric kidney diseases [48]. In addition, suPAR levels predicted steroid-responsiveness of FSGS. There was no association of suPAR levels with eGFR, but only patients with eGRF >40 mL/min were included in the study and therefore these results need to be interpreted with caution.

Since the original description of suPAR as a potential causal factor in primary FSGS, many researchers have tested suPAR levels in human adult and pediatric cohorts with conflicting results [41, 42, 44, 49, 50]. For example, Meijers et al. measured suPAR levels in control patients with CKD (476) and biopsy-proven FSGS patients (44) [42]. Multivariate analysis revealed a strong inverse association of suPAR with eGFR and serum albumin, while there was a positive association with age and CRP. No differences in suPAR levels were identified amongst FSGS and control patients. In the Nephrotic Syndrome Study Network (NEPTUNE including adults and children) cohort, suPAR levels were analyzed in 241 patients with FSGS (60), MCD (104), IgA nephropathy (57), and MN (82) [41]. In this cohort of proteinuric kidney disease, suPAR levels inversely correlated with eGFR and proteinuria in all disease groups. Multivariate linear regression depicted that plasma suPAR concentration was not associated with FSGS after adjustment of eGFR. With regard to the clinical endpoints in the NEPTUNE cohort, plasma suPAR levels did not predict the occurrence of end-stage renal disease (ESRD), 50% loss of eGFR, and complete or partial remission after adjustment of eGFR or proteinuria. Wada et al. confirmed the relationship of suPAR levels and eGFR in a Japanese cohort with primary glomerular diseases including FSGS [44]. In patients with eGFR >60 mL/min/1.73 m^2^, suPAR levels did not discriminate primary FSGS from other glomerular pathologies. Even though Huang et al. reported elevated suPAR concentrations in FSGS in a Chinese cohort, suPAR levels did not differentiate between primary and secondary FSGS [49]. In addition, several pediatric cohort studies did not confirm the initial reports that serum or plasma suPAR levels could serve as a biomarker for primary FSGS [43, 51, 52].

Taken together from the evidence presented above, plasma and serum suPAR levels do not discriminate primary FSGS from other proteinuric kidney diseases. However, suPAR seems to be a biomarker for reduced renal function. Due to its molecular size (25–50 kDa), suPAR is probably filtered by the glomerulus. Reduced eGFR will lead to a reduction of filtered suPAR resulting in potentially increased serum and plasma levels of suPAR. Nothing so far is known about the tubular processing of suPAR.

Evidence points towards a role of suPAR as a microinflammatory marker in FSGS [42]. However, in a CKD population, suPAR seems to have additional prognostic value beyond conventional microinflammatory markers [40]. Elevated suPAR levels predict development of CKD in patients with cardiovascular disease [45]. However, in patients with preserved renal function, elevated suPAR levels cannot be explained by the theory of reduced suPAR filtration [53]. Further studies will need to clarify the role of suPAR as a biomarker in patients with preserved renal function.

#### 2.1.4. Does suPAR Cause Podocyte Injury and Proteinuria in FSGS?

In the studies of Wei et al., the hypothesis that suPAR causes FSGS derived from* in vitro* and* in vivo* experiments [46]. As described above, FSGS sera led to robust staining with an AP5 antibody indicating activated *α*_*v*_ *β*_3_-integrin in human podocytes and glomeruli as the pathomechanism of primary FSGS. In contrast, Yu et al. reported that *α*_*v*_ *β*_1_ was the essential integrin in five patients with FSGS (one with primary FSGS and four with recurrent FSGS) [54]. Mechanistically, B7-1 (CD80) deactivated *α*_*v*_ *β*_1_ but not *α*_*v*_ *β*_3_-integrin in podocytes of these patients who were also glucocorticoid and rituximab resistent. Abatacept, a costimulatory inhibitor of B7-1, induced remission in all of these patients. Interestingly, some biopsy specimen from patients with other proteinuric kidney diseases had positive B7-1 staining indicating that B7-1 might be a biomarker of podocyte injury and could identify patients that may benefit from therapy with abatacept [54]. However, conflicting results have also been published by Benigni et al. and Delville et al. [55, 56].

Following the original article of Wei et al., three different mouse models confirmed further the hypothesis that suPAR caused proteinuria in mice. In* Plaur *−*/*− mice, infusion of recombinant suPAR (recombinant mouse suPAR-Fc) caused proteinuria and these mice became protected from LPS induced proteinuria. Furthermore, wild-type mice with transplanted kidneys from* Plaur *−*/*− mice were challenged with LPS and developed proteinuria. Lastly, wild-type mice that were treated with gene transfer (sPlaur_WT_ or sPlaur_E134A_ mutant potentially defective of *β*_3_-integrin binding) were analyzed for protection from LPS induced proteinuria. Mice that received gene transfer of the defective integrin binding suPAR mutant were protected. In line with these results, Alfano et al. showed that high dosages of suPAR (recombinant mouse suPAR-Fc) induced proteinuria in* Plaur *−*/*− mice [57]. Providing further insights into the potential pathomechanism of suPAR action, Alfano et al. described that suPAR decreased nephrin expression in podocytes via suppression of Wilms tumor-1 (WT-1) transcription factor. Interestingly, only the full-length suPAR molecule interacted with *β*_3_-integrin and caused podocyte damage. Cleaved suPAR molecules were not able to activate *β*_3_-integrin. Delville et al. revealed that suPAR (recombinant human suPAR) exacerbated proteinuria in an anti-CD40 antibody mediated proteinuria model [58].

In contrast, Spinale et al. did not find proteinuria after 24 h in wild-type mice after administration of recombinant suPAR (recombinant mouse suPAR-Fc) even though high suPAR levels were detected [41]. In addition, ectopic expression of the full-length suPAR (*D*_I_–*D*_III_) molecule from the liver did not induce proteinuria for 44 days despite elevated suPAR levels. Similarly, Cathelin et al. were not able to demonstrate that short-term and prolonged administration of suPAR (recombinant mouse suPAR-Fc and monomeric mouse uPAR produced in S2-cells) caused proteinuria in wild-type mice [59].

This conflicting data can partly be explained by the different genetic backgrounds of the mice (Plaur −/− versus WT) investigated in the different studies. In addition, Wei et al. used a splice variant of mouse suPAR containing a retained intron 4 in their gene transfer experiments. If not spliced out, intron 4 would have led to a premature stop within uPAR domain 2 [41]. The expression of this suPAR variant seems to be rare and the homologous splice variant has not yet been identified in humans [41].

Many questions about the potential causal role of suPAR for podocyte damage remain. Due to the conflicting data at the moment, more evidence is needed that circulating suPAR causally leads to podocyte damage in primary FSGS patients.

### 2.2. Cardiotrophin-Like Cytokine Factor-1 (CLCF-1)

CLCF-1 is a member of the IL-6 family of cytokines with a predicted molecular weight of 22 kDa [60]. CLCF-1 is secreted and forms heterodimers with either cytokine receptor like factor 1 (CRLF1) or soluble ciliaric neurotrophic receptor alpha (sCNTFR*α*) resulting in composite cytokine [61].

#### 2.2.1. CLCF-1 in FSGS

The identification of CLCF-1 as a potential permeability factor in primary FSGS was the result of studying FSGS plasma for more than 20 years by Savin's group [11, 13, 17, 18, 60–65]. Through systematic investigation of the biochemical characteristics of the active fraction of FSGS plasma, Savin's group was able to isolate the permeability factor by galactose affinity chromatography and mass spectrometry [18]. After dialyzation of the eluate from the galactose column, the permeability factor was identified in the fraction <30 kDa [18]. Finally, CLCF-1 was found in the active fraction of plasma from patients with recurrent FSGS [18, 65]. The concentration of CLCF-1 in plasma from patients with recurrent FSGS was up to 100 times higher than in controls [65]. In preliminary studies, the concentration of CLCF-1 in healthy subjects was only 100 pg/mL [60]. Therefore, available assays to measure CLCF-1 are not sensitive enough to detect CLCF-1 levels in patient samples at the moment [60]. In addition, CLCF-1 levels have not been investigated in other disease states or in the urine of FSGS patients so far [60]. In the future, assays for CLCF-1 detection need to be developed. Evaluation of CLCF-1 levels in clinically well defined cohorts (e.g., NEPTUNE, FSGS CT) is necessary to prove CLCF-1's pathophysiological role only in primary FSGS. If confirmed, CLCF-1 is an excellent candidate for therapy as no essential role for CLCF-1 is described after fetal development [60]. Antibodies targeting CLCF-1 or its receptors could be potential future and individualized treatment strategies.

#### 2.2.2. Does CLCF-1 Cause Podocyte Injury and Proteinuria in FSGS?

Several years ago, Savin's group developed an* in vitro* assay to study glomerular permeability [62]. In isolated rat glomeruli, an isotonic albumin oncotic solution was replaced by a solution with a lower albumin concentration. This led to an increase in glomerular size through swelling if the permeability barrier was intact. Incubation of the glomeruli with FSGS sera led to a decrease of glomerular size compared to control glomeruli. This indicated a disruption of the oncotic gradient through an increase in glomerular permeability. Permeability to albumin (*Palb*) was expressed as 1 minus the difference in glomerular size. CLCF-1 mimicked the effects of FSGS plasma on* Palb,* while a CLCF-1 antibody abolished this effect. In addition, CLCF-1 decreased nephrin expression in glomeruli and podocytes. Incubation of murine podocytes with CLCF-1 disrupted the actin cytoskeleton in a time and concentration dependent manner and led to a motile phenotype of the podocytes [60]. More recently, recombinant monomeric human CLCF-1 increased* Palb* in isolated rat glomeruli [61] as well as albuminuria in mice after acute and chronic infusion [60]. However, heterodimers of CLCF-1 with CRLF-1 blocked the increase in* Palb* from FSGS sera [61]. In addition, inhibitors of the Jak-Stat3 signaling pathway abolished the increase in* Palb* from CLCF-1 or FSGS sera [60, 61].

As described above, CLCF-1 was found in the active fraction of FSGS sera and was isolated by galactose affinity chromatography. Furthermore, application of galactose blocked the increase of Palb by FSGS sera [65]. However, several case reports have shown conflicting results on the treatment of FSGS patients with galactose [66–68]. Recently, the FONT II trial was published as a phase I/II open-label randomized controlled trial. The trial compared standard conservative therapy (SCT) versus SCT plus adalimumab (antibody against tumor necrosis factor-*α*/TNF-*α*) versus SCT plus galactose [64]. Patients with biopsy-proven primary FSGS or genetic FSGS, with proteinuria of >1 g/g and eGFR >40 mL/min/1.73 m^2^, were included. The patients received therapy over 26 weeks and the primary end point was a 50% reduction in proteinuria with stable GFR. Of the 21 patients included in the study, 7 received SCT plus galactose. Three out of seven patients met the primary end point. No improvement was noted with treatment of SCT plus adalimumab. Even though primary FSGS is a rare disease, further and larger studies are needed to confirm the potential benefit from galactose treatment in FSGS.

Taken together, the identification of CLCF-1 as a potential circulating permeability factor is very promising. However, its pathophysiological role needs to be validated in well characterized patient cohorts and by different research groups in the future.

### 2.3. Anti-CD40 Antibodies

The costimulatory molecule CD40 is a member of the TNF receptor superfamily [69]. CD40 is an important molecule in immunity and inflammation. It is expressed in various tissues especially on the surface of antigen presenting cells (APCs), macrophages/monocytes, and dendritic cells [69]. CD40 is also expressed in endothelial and epithelial cells. CD40 ligand binds to CD40 and is expressed also in many different cell types such as immunological, endothelial, and epithelial cells [69]. CD40 ligand activates endothelium and leads to increased expression of chemokines, metalloproteases, uPA, and suPAR [58].

#### 2.3.1. Anti-CD40 Autoantibodies in FSGS

Delville et al. described the identification of a panel of autoantibodies in recurrent FSGS before transplantation [58]. Using array data and an enzyme linked immunosorbent assay (ELISA), pretransplant sera from 20 patients with FSGS and biopsy-proven FSGS were analyzed [58]. 10 patients had disease recurrence in the first year of transplantation (recurrent FSGS) and 10 had no recurrence of proteinuria or histological disease after transplantation (nonrecurrent FSGS). IgG profiles from the sera of recurrent and nonrecurrent FSGS varied significantly and, after validation with different tools, autoantibodies against CD40 were the most promising antibodies to pursue further.

#### 2.3.2. Do Anti-CD40 Autoantibodies Cause Podocyte Injury and Proteinuria in FSGS?

CD40 is expressed in human cultured podocytes and its expression cannot be induced by challenging* in vitro* [58]. However, in patients with FSGS, CD40 was detected in glomeruli from recurrent FSGS patients. Interestingly, the autoantibodies against CD40 did not recognize human CD40 and anti-CD40 antibody reactive regions differed between recurrent and nonrecurrent FSGS sera. Even though autoantibodies against CD40 from recurrent FSGS sera did not detect recombinant human CD40, purified CD40 autoantibodies from recurrent FSGS sera disrupted the podocyte (human) actin cytoskeleton* in vitro*. This finding points to a posttranslational modification of the CD40 molecule* in vivo* that is necessary for detection with CD40 autoantibodies. The data of Delville et al. further suggested that suPAR-*β*_3_-integrin pathway could be involved. In addition, injection of anti-CD40 antibodies from recurrent FSGS patients into wild-type mice was not sufficient to cause robust albuminuria. However, if full recombinant suPAR was coadministered, albuminuria developed. An antibody against suPAR or a small molecule targeting the activation of *α*_*v*_ *β*_3_-integrin blocked the effect of CD40/suPAR. There was no increase in glomerular permeability in* CD40 *−*/*− or wild-type animals injected with recombinant CD40. The authors concluded that CD40 autoantibodies have a pathogenetic role in the development of recurrent FSGS potentially through interaction with suPAR.

The size of IgG antibodies is approximately 150 kDa [70]. The size of intact CD-40 autoantibodies therefore contradicts previous findings that the active fraction of FSGS sera was smaller than 30–50 kDa [17].

Besides these exciting findings, the role of CD40 antibodies in human disease needs to be validated. Anti-CD40 blocking antibodies (ASKP1240 or lucatumumab) are already commercially available and could become potential treatment options tested in clinical trials [71].

## 3. Conclusion

The clinical evidence presses for the existence of circulating permeability factors in primary FSGS. Some molecules have been proposed but have not finally proven their pathogenic role. So far, none of the proposed molecules have been validated by different research groups in different FSGS disease models. We expect additional promising data for known and novel candidates in near future. Hopefully, this will enable us to treat patients with primary FSGS individually based on their pathogenic circulating factor.

## Figures and Tables

**Table 1:** Circulating permeability factors in primary FSGS: summary of proposed candidates.

Circulating factor	Molecular weight (kDa)	Experimental findings	Clinical data for FSGS and CKD
suPAR	25–50	Administration of suPAR caused albuminuria in uPAR −/− mice [46], however not in WT mice [41, 59] Activation of podocytic *α*_*v*_ *β*_3_-integrin leading to cytoskeletal rearrangement [46] Decrease of nephrin expression via suppression of WT-1 [57]	suPAR levels are inversely correlated with eGFR, no discrimination of primary FSGS to other proteinuric diseases [41, 42, 44, 49, 50] suPAR seems to be a microinflammatory marker in FSGS [40] suPAR predicts CKD in a cardiovascular cohort [45] Significance of suPAR levels as a biomarker for FSGS in patients with preserved renal function unclear [53]

CLCF-1	22	Binds to galactose columns [18, 61, 65] and galactose blocked increase in glomerular permeability by FSGS sera [65] Administration of CLCF-1 increases glomerular permeability and proteinuria in mice [60] Decreases nephrin expression and disrupts the podocytic cytoskeleton [60] Inhibitors of the Jak/Stat3 pathway abolish CLCF-1 and FSGS sera effects [61]	Concentration of CLCF-1 in FSGS patients up to 100-fold higher than in controls, however available assay too insensitive at the moment [60] Current data do not support therapy of FSGS patients with galactose [64] Due to measurement difficulties not tested in FSGS cohorts yet

CD40 autoantibodies	150	Expressed in glomeruli from FSGS patients [58] Disrupt podocyte actin cytoskeleton [58] Injection of CD40 autoantibodies leads to albuminuria only if recombinant suPAR is coadministered [58] Administration of CD40 autoantibodies does not increase glomerular permeability in CD40 −/− mice [58]	Identified in autoantibody panel from sera of patients with recurrent FSGS [58]

suPAR, soluble urokinase plasminogen activator receptor; CLCF-1, cardiotrophin-like cytokine factor-1; WT, wild-type; WT-1, Wilms tumor-1; FSGS, focal segmental glomerular sclerosis; eGFR, estimated glomerular filtration rate; Jak, Janus-kinase; Stat3, signal transducer and activator of transcription 3.
